# Case Report: Primary and Acquired Resistance Mechanisms of Nimotuzumab in Advanced Esophageal Squamous Cell Carcinoma Revealed by Targeted Sequencing

**DOI:** 10.3389/fonc.2020.574523

**Published:** 2020-10-29

**Authors:** Dantong Sun, Weihua Yan, Hua Zhu, Qiaoling Liu, Helei Hou

**Affiliations:** ^1^ Precision Medicine Center of Oncology, The Affiliated Hospital of Qingdao University, Qingdao, China; ^2^ Department of Pathology, The Affiliated Hospital of Qingdao University, Qingdao, China; ^3^ Department of Medical Oncology, Qingdao West Coast New Area Central Hospital, Qingdao, China

**Keywords:** nimotuzumab, resistance, PIK3CA, mTOR, PD-L1, esophageal squamous cell carcinoma

## Abstract

Esophageal squamous cell carcinoma (ESCC) is a deadly disease with a low 5-year survival rate. Anti-epidermal growth factor receptor (EGFR) therapy has been widely used in the treatment of malignancies, and chemotherapy regimens that include nimotuzumab have been confirmed to have satisfactory efficacy among esophageal carcinoma (EC) patients. However, a subpopulation of patients may develop resistance to nimotuzumab. Here, we report an advanced ESCC patient who experienced hyperprogressive disease induced by immune checkpoint inhibitors and was then treated with a chemotherapy regimen containing nimotuzumab. NGS examination of this patient demonstrated that *PIK3CA* mutation and a *RICTOR* amplification might participate in primary and acquired resistance to nimotuzumab, respectively, *via* the PI3K/AKT/mTOR signaling pathway.

## Background

Esophageal carcinoma (EC), which is the sixth leading cause of cancer-related mortality, affects more than 450,000 people worldwide. Patients suffering from EC may have a poor prognosis as a result of the stage at diagnosis and the metastatic properties of EC (1, 2). Esophageal squamous cell carcinoma (ESCC) is the predominant pathological type of EC (3), especially in eastern Asia, and is associated with a series of risk factors, including smoking status, alcohol consumption, improper eating habits, and poor nutrition (4).

ESCC is a deadly disease, with a 5-year survival rate of approximately 10% that requires treatment, including surgical operation, systemic chemotherapy, and radiotherapy. With the development of targeted sequencing, targeted therapy has been widely applied in the treatment of ESCC. Protein overexpression of epidermal growth factor receptor (EGFR) and *EGFR* amplification has been observed in many ESCC patients (5) and is related to a poor prognosis in both progression-free survival (PFS) and overall survival (OS) (6). Nimotuzumab (h-R3) is a humanized anti-EGFR monoclonal antibody that has demonstrated efficacy in EGFR-positive ESCC patients, as verified by multiple clinical trials (7–9). Nimotuzumab combined with paclitaxel plus cisplatin (TPN regimen), cisplatin plus 5-FU (PF + N) or concurrent chemoradiotherapy (nimotuzumab plus CCRT) all showed promising efficacy and tolerable safety in treating ESCC. In addition, monotherapy with nimotuzumab followed by radiotherapy yielded encouraging OS, PFS, and locoregional control (LC) (10). In another phase III clinical trial, Jing et al. (11) confirmed that nimotuzumab demonstrated better efficacy than did cetuximab (C225), a recombinant human/mouse chimeric EGFR monoclonal antibody. Nimotuzumab showed a significantly longer median PFS than cetuximab and a similar incidence of grade 3 or worse adverse events (AEs).

Regrettably, not all patients harboring *EGFR* amplification or EGFR protein overexpression respond well to nimotuzumab, while primary and acquired resistance to nimotuzumab from monotherapy or combined regimens can be observed in multiple studies on ESCC (7–10, 12). However, the mechanisms of primary and acquired resistance to nimotuzumab require further studies.

## Methods for NGS Assay and Disease Evaluation

As described in our previous study (13), the tissue samples of the patient in this report were examined by next-generation sequencing (NGS) assay using three versions of a capture-based targeted sequencing panel. Tissue for the first NGS assay (2018-07) was acquired from the primary tumor during surgical resection, while metastatic tissue in the lung was acquired *via* percutaneous lung puncture and sent for the second NGS assay (2019-02). Blood samples were also acquired for each NGS assay. Genomic alterations were detected in 17 genes, including 16 genes altered before nimotuzumab treatment and 15 genes altered after the treatment. For disease evaluation, we used the Response Evaluation Criteria in Solid Tumors 1.1 (RECIST 1.1) to conduct the assessment of disease status *via* 64-slice spiral CT scan (HITACHI, JAPAN). Blood samples from the patient were sent for tumor marker examination in the clinical laboratory at our hospital. The patient in this report gave his consent for the publication of the report.

## Patient Story

**Figure 1:**
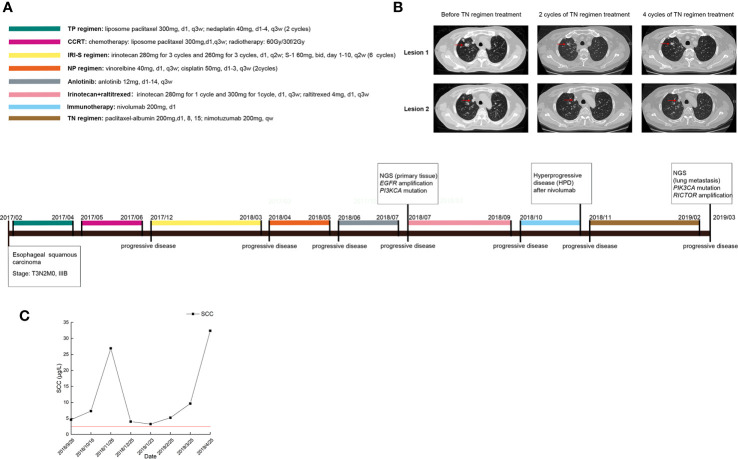
Clinical data of the patient during the treatment. **(A)** The timeline of treatments for a patient with advanced esophageal squamous carcinoma; **(B)** CT scans before and after nimotuzumab treatment; **(C)** Changes in tumor marker levels during treatment.

In July 2018, a 52-year-old male with ESCC visited our hospital after multiple lines of treatment. The patient was first diagnosed with stage IIIB ESCC *via* postsurgical pathology on February 7^th^, 2017. The patient then received 2 cycles of the TP chemotherapy regimen and CCRT after surgery and remained stable until the first instance of recurrence on December 12^th^, 2017. First-line chemotherapy and further antiangiogenetic therapy were administered to the patient but were not effective. Therefore, the patient came to our hospital and received treatment, the detailed timeline of which is shown in **Figure 1A**. According to the CheckMate-032 study (14), nivolumab demonstrated clinically meaningful antitumor activity in patients with chemotherapy-refractory esophagogastric cancer. Considering that this patient had experienced PD after multiple lines treatment regimens, he was then treated with nivolumab after acquired consent. Therefore, the patient received 200 mg (3 mg/kg, q2w) of nivolumab on October 18^th^, 2018. However, the patients developed a hyperprogressive disease (HPD). Upon disease assessment, the metastatic lesions in the lung grew more than 50% compared with baseline within 1 month *via* the evaluation of CT scans, and the patient suffered severe immune-related pneumonia. After symptomatic and supportive treatment, the patient started a regimen of nimotuzumab combined with chemotherapy. After two cycles of the TN (nab-paclitaxel + nimotuzumab) regimen, the response was declared a partial response (PR), but after four cycles of this regimen, the patient showed progressive disease (PD). The sizes of two metastatic lesions in the lung increased more than 20% compared with the most recent CT scan (lesion 1: 23%; lesion 2: 24%; average: 23.4%), as shown in **Figure 1B**, and the levels of squamous cell carcinoma (SCC) antigen, a tumor marker, rose drastically, as shown in **Figure 1C**. Dynamic NGS was performed before and after nimotuzumab treatment, and the results are summarized in **Table 1**. NGS assays of this patient revealed that *EGFR* amplification and the p.E545K mutation of *PIK3CA* were present in his peripheral blood before nimotuzumab treatment. According to the NGS examination after nimotuzumab treatment, amplification of *rapamycin-insensitive companion of mammalian target of rapamycin* (*RICTOR*) was detected, which indicated activation of the mTOR signaling pathway, while amplification of *EGFR* was not detected upon examination.

**Table 1:** Results of NGS assays for the ESCC patient.

Gene	Before nimotuzumab (2018-07)		Post nimotuzumab (2019-02)	
	Abundance	Alteration type	Abundance	Alteration type
***EGFR***	CN: 4.55	Amplification		
***TP53***	44.60%	Exon 6, p.Y220C, missense	34.52%	Exon 6, p.Y220C, missense
***NOTCH1***	57.73%	Exon 6, p.C359Y, missense	21.44%	Exon 6, p.C359Y, missense
***HLA-A***	55.20%	Exon 5, p.S337P, missense	37.98%	Exon 5, p.S337P, missense
***PPM1D***	0.57%	Exon 6, non-sense		
***EP300***	30.14%	Exon 28, p.W1509R, missense	39.72%	Exon 28, p.W1509R, missense
***ARID1A***	0.83%	Exon 7, non-sense	13.79%	Exon 7, non-sense
***ASXL1***	1.74%	Exon 12, p.P1035S, missense	11.89%	Exon 12, p.P1035S, missense
***PIK3CA***	1.94%	Exon 10, p.E545K, missense	13.09%	Exon 10, p.E545K, missense
***HSD3B1***	1.60%	Exon 4, p.Y181C, missense	14.94%	Exon 4, p.Y181C, missense
***PTCH1***	1.93%	Exon 18, p.E970K, missense	13.47%	Exon 18, p.E970K, missense
***FAT3***	2.50%	Exon 9, p.T1874A, missense	10.90%	Exon 9, p.T1874A, missense
***LRP1B***	2.24%	Exon 18, p.D2621H, missense	19.19%	Exon 18, p.D2621H, missense
***FGF10***	1.34%	Exon 10, p.V77F, missense	7.06%	Exon 10, p.V77F, missense
***SMAD4***	1.29%	Exon 11, non-sense	16.31%	Exon 11, non-sense
***TERT***	1.22%	Exon 9, p.R858Q, missense	8.03%	Exon 9, p.A858G, missense
***RICTOR***			CN: 3.4	Amplification

1. Abundance: the proportion of cells harboring the corresponding genomic alterations in the tumor tissue.

2. CN, copy number.

## Discussion

Anti-EGFR monoclonal antibodies (mAbs) have been widely used to treat malignancies, and patients have benefited from anti-EGFR mAbs such as cetuximab and nimotuzumab. However, resistance to anti-EGFR mAbs has diminished the available options for patients. Multiple studies have focused on the mechanisms of primary and acquired resistance to cetuximab. Since anti-EGFR mAbs block the activation of the EGFR signaling pathway, alterations in the expression and activity of EGFR signaling pathway-related genes may play important roles. *PIK3CA* mutations result in the persistent phosphorylation of Akt1 and overactivate the EGFR signaling pathway, which is related to primary resistance to cetuximab (15). Recently, studies have verified the relationship between *PIK3CA* mutation and primary cetuximab resistance (15–17). After phosphorylation of Akt1 and activation of the EGFR signaling pathway, *PIK3CA* mutations can lead to cetuximab resistance *via* activation of the downstream mTOR signaling pathway (18). Extraordinarily, the p.E545K mutation of *PIK3CA* enhances the binding ability to EGFR and significantly activates the downstream Akt signaling pathway (19). The mechanism of resistance to cetuximab seems clear, but no study has provided a clue as to whether nimotuzumab shares this resistance mechanism. We provided this case report to demonstrate the efficacy and safety of nimotuzumab in ESCC patients who experienced immunotherapy-related HPD and the potential mechanism of nimotuzumab resistance.

The patient here developed HPD and severe pneumonia and had poor fundamental pulmonary function, but nimotuzumab combined with nab-paclitaxel (TN regimen) demonstrated its advantage in safety. The four-cycle TN regimen was well tolerated by this patient, and the only Grade 3 AE reported was rash acneiform, which was located on his back and face. A PR was declared after the first two cycles of the TN regimen, demonstrating the efficacy of nimotuzumab combined with chemotherapy, even after HPD. However, the response to the TN regimen lasted only four cycles, which was less than 4 months. Given that nimotuzumab is a mAb for EGFR, the p.E545K mutation of *PIK3CA* in this patient might have led to constitutive Akt phosphorylation, and activation of the EGFR signaling pathway is strongly associated with primary resistance to nimotuzumab and a short time to treatment failure (TTF). Meanwhile, based on the NGS results before and after nimotuzumab treatment, we proposed that activation of the phosphoinositide-3-kinase (PI3K)/AKT/mTOR signaling pathway is the potential mechanism of primary and acquired resistance to nimotuzumab. Nimotuzumab combined with nab-paclitaxel might be an alternative choice for ESCC patients who experience HPD with tolerance safety. *PIK3CA* mutation and *RICTOR* amplification may participate in primary and acquired resistance to nimotuzumab, respectively, *via* the PI3K/AKT/mTOR signaling pathway. Further treatment with alpelisib (BYL719) (20) or rapalagos (21), which target the mTOR pathway, might benefit ESCC patients harboring mutations or genetic alterations that increase the activation of the PI3K–AKT/mTOR signaling pathway.

## Ethics Statement

The studies involving human participants were reviewed and approved by the Affiliated Hospital of Qingdao University. The patients/participants provided their written informed consent to participate in this study. Written informed consent was obtained from the individual(s) for the publication of any potentially identifiable images or data included in this article.

## Author Contributions

Conception/Design: HH. Provision of study material or patients: HH, WY and HZ. Collection and/or assembly of data: DS, QL, and HZ. Data analysis and interpretation: DS, QL, and HZ. Manuscript writing: HH and DS. All authors contributed to the article and approved the submitted version.

## Funding

Special funding from the Qilu Sanitation and Health Leading Talents Cultivation Project (to HH.)

## Conflict of Interest

The authors declare that the research was conducted in the absence of any commercial or financial relationships that could be construed as a potential conflict of interest.
